# No Effect of Remote Ischemic Conditioning Strategies on Recovery from Renal Ischemia-Reperfusion Injury and Protective Molecular Mediators

**DOI:** 10.1371/journal.pone.0146109

**Published:** 2015-12-31

**Authors:** Casper Kierulf-Lassen, Marie Louise Vindvad Kristensen, Henrik Birn, Bente Jespersen, Rikke Nørregaard

**Affiliations:** 1 Department of Clinical Medicine, Aarhus University, Aarhus, Denmark; 2 Department of Renal Medicine, Aarhus University Hospital, Aarhus, Denmark; 3 Department of Biomedicine, Aarhus University, Aarhus, Denmark; University of Pecs Medical School, HUNGARY

## Abstract

Ischemia-reperfusion injury (IRI) is the major cause of acute kidney injury. Remote ischemic conditioning (rIC) performed as brief intermittent sub-lethal ischemia and reperfusion episodes in a distant organ may protect the kidney against IRI. Here we investigated the renal effects of rIC applied either prior to (remote ischemic preconditioning; rIPC) or during (remote ischemic perconditioning; rIPerC) sustained ischemic kidney injury in rats. The effects were evaluated as differences in creatinine clearance (CrCl) rate, tissue tubular damage marker expression, and potential kidney recovery mediators. One week after undergoing right-sided nephrectomy, rats were randomly divided into four groups: sham (n = 7), ischemia and reperfusion (IR; n = 10), IR+rIPC (n = 10), and IR+rIPerC (n = 10). The rIC was performed as four repeated episodes of 5-minute clamping of the infrarenal aorta followed by 5-minute release either before or during 37 minutes of left renal artery clamping representing the IRI. Urine and blood were sampled prior to ischemia as well as 3 and 7 days after reperfusion. The kidney was harvested for mRNA and protein isolation. Seven days after IRI, the CrCl change from baseline values was similar in the IR (δ: 0.74 mL/min/kg [-0.45 to 1.94]), IR+rIPC (δ: 0.21 mL/min/kg [-0.75 to 1.17], *p* > 0.9999), and IR+rIPerC (δ: 0.41 mL/min/kg [-0.43 to 1.25], *p* > 0.9999) groups. Kidney function recovery was associated with a significant up-regulation of phosphorylated protein kinase B (pAkt), extracellular regulated kinase 1/2 (pERK1/2), and heat shock proteins (HSPs) pHSP27, HSP32, and HSP70, but rIC was not associated with any significant differences in tubular damage, inflammatory, or fibrosis marker expression. In our study, rIC did not protect the kidney against IRI. However, on days 3–7 after IRI, all groups recovered renal function. This was associated with pAkt and pERK1/2 up-regulation and increased HSP expression at day 7.

## Introduction

Ischemia-reperfusion injury (IRI) is the leading cause of acute kidney injury (AKI) in a variety of clinical settings, e.g. renal transplantation, cardiovascular surgery, and hypovolemic/septic shock [1]. AKI may progress to chronic kidney disease due to tubular epithelial cell damage, endothelial dysfunction, and abnormal repair. Abnormal repair can lead to renal fibrosis due to pro-fibrotic factors such as transforming growth factor-β1 (TGF-β1), the generation of excess extracellular matrix, and chronic hypoxia [1–3]. Thus, the development of strategies to diminish IRI-induced damage is crucial. In experimental models, ischemic conditioning (IC) has been shown to confer protection to the kidney against IRI [4,5]. One or more brief episodes of sub-lethal, intermittent ischemia and reperfusion (IR) may be produced locally in the kidney (local IC) or in a remote tissue (rIC) [4,5]. Local IC mobilises a multitude of pro-survival pathways in the kidney [6]. However, in contrast to local IC, few attempts have been made to investigate the underlying renal molecular mechanisms of rIC and whether the renal responses to different rIC types, e.g. before IRI (remote ischemic preconditioning; rIPC) or during IRI (remote ischemic perconditioning; rIPerC), are similar [6].

Intracellular mitogen-activated kinases (MAPK), including Akt and ERK1/2, are members of the reperfusion injury survival kinase (RISK) pathway [7]. The activation of this pathway is reported to be particularly important during early reperfusion to confer protection against IRI [8]. In a delayed type of protection, either local or remote IPC recurs 12–72 hours after the conditioning stimulus [9,10]. This is considered dependent on de novo protein synthesis, including heat shock proteins (HSPs), manganese superoxide dismutase (MnSOD), inducible nitric oxide synthase (iNOS), cyclooxygenase-2, and aldose reductase [9]. It has been speculated that one or more of these proteins are capable of reactivating the RISK pathway, although the exact mechanisms are not completely clear [9]. Interestingly, ERK1/2 and Akt are persistently activated several days after ischemia and possibly participate in the long-term repair process after IRI independent of IC [11,12]. It is not clear whether rIC is capable of increasing and maintaining high levels of cytoprotective proteins in the kidney several days after the ischemic insult.

This study was designed to evaluate the recovery capacity of the kidney after IRI and investigate whether rIPC or rIPerC offer additional protective effects against IRI-induced functional impairment, tissue inflammation, and fibrosis via the up-regulation of established cytoprotective proteins. We observed that animals subjected to 37 minutes of unilateral ischemia recovered kidney function at day 7 post-ischemia, and this was associated with an up-regulation of the protein levels of pAkt, pERK1/2, and HSPs as well as an increased iNOS mRNA level. We did not detect any obvious renal effects of rIC.

## Materials and Methods

### Experimental Animals

The study was approved by The Ministry of Food, Agriculture and Fisheries, Animal Experiments Inspectorate (approval no. 2013−15−2934−00810). All procedures adhered to the guidelines published by the National Institutes of Health and the European Convention for the Protection of Vertebrate Animals Used for Experimental and Other Scientific Purposes. Housing conditions and husbandry practices were identical across the control and experimental groups. Adult male rats (*Rattus norvegicus*, Wistar) weighing 240–290 g were supplied by Taconic Europe (Ry, Denmark) and housed in cages pairwise under standard conditions (12 h:12 h light-dark cycle, constant temperature of 21 ± 2°C, and humidity of 55 ± 2%). The rats had free access to tap water and standard rodent chow at all times. The rats were cared for daily and monitored for pain and distress between and after the procedures using a general distress scoring sheet [13]. The approval for this experiment included a protocol for early euthanization of animals who exhibited signs of severe illness and contained the following humane endpoints; poor welfare with insufficient food and water intake, constant piloerection, signs of infections, bleeding from wounds, and weight loss over 20%.The rats were allowed to acclimate at least 1 week prior to the surgical procedures.

### Study Design

**Fig 1:**
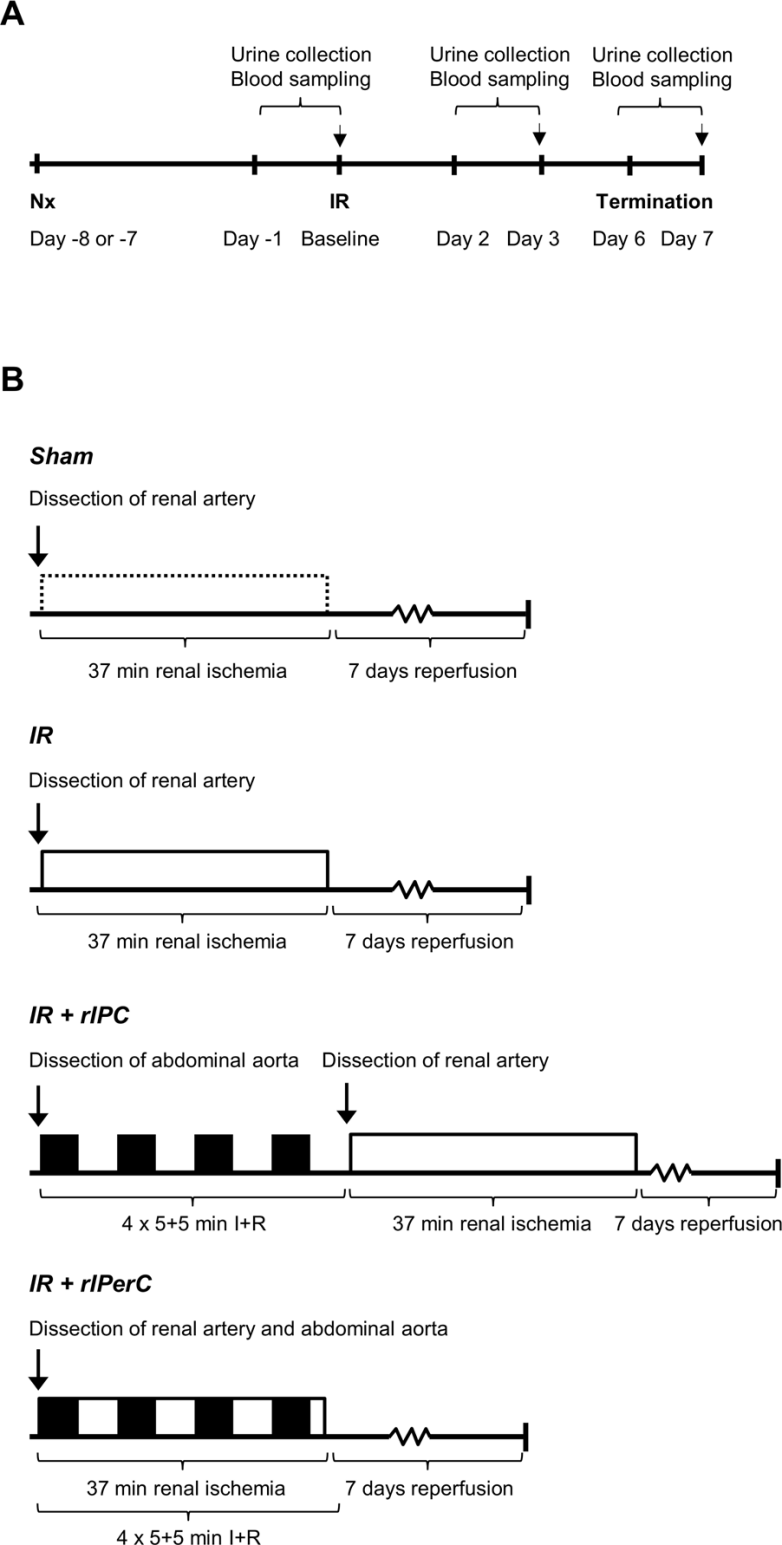
The study design and the four experimental groups. A) All animals underwent right nephrectomy 7–8 days prior to the renal ischemia-reperfusion injury or sham operation. Urinary creatinine clearance was determined prior to renal ischemia-reperfusion (IR; termed baseline) and days 3 and 7 post-operatively. B) The remote ischemic conditioning stimulus was applied as four cycles of 5 minutes of ischemia and 5 minutes of reperfusion by clamping the infrarenal aorta either before (remote ischemic preconditioning; rIPC) or during (remote ischemic perconditioning; rIPerC) 37 minutes of unilateral renal ischemia.

All animals underwent right nephrectomy 7–8 days prior to the renal IRI or sham operation. The rats were then randomised into the following groups: sham (n = 7), IR (n = 10), IR+rIPC (n = 10), and IR+rIPerC (n = 10). The randomization took place in the morning of each day prior to ischemia or sham operation, i.e. 7–8 days after the unilateral nephrectomy procedure. The animals were individually randomized, and allocated to the different treatment groups by drawing lots. In order to avoid day-to-day variations in perioperative conditions to result in any systematic differences in the groups, we also used block randomization. Each day all 3–4 groups were represented in the drawing pool in a number proportionate to their final group size. The sequence of the animals was determined by the order in which they were drawn. No further measures of randomization were found to be feasible during the rest of the experiments. The study was not blinded.The IR, IR+rIPC, and IR+rIPerC groups were subjected to 37 minutes of warm renal ischemia by clamping of the left renal artery. Urine was collected for 24 hours in metabolic cages before (defined as baseline) as well as 2 and 6 days after the IRI (Fig 1A). Ethylenediaminetetraacetic acid (EDTA)-treated blood was sampled at the end of each urine collection period: i.e. baseline and at days 3 and 7. The primary outcome, i.e. urinary creatinine clearance (CrCl) rate, was calculated as 24-hour urine creatinine excretion divided by plasma creatinine (pCr). The rats were allowed to acclimate in the metabolic cages 24 hours prior to the actual urine collection periods except at the end of the study. The rIC stimulus was induced using a 4×5+5–minute IR protocol: i.e. 5 minutes of ischemia followed by 5 minutes of reperfusion repeated four times either prior to (IR+rIPC group) or during (IR+rIPerC) IRI (Fig 1B). The rIC was applied by infrarenal aorta clamping above the bifurcation. Pilot studies were performed to establish an appropriate ischemia time (30, 37, or 45 minutes) based on the increase in pCr and survival. A choice of 37 minutes of ischemia was chosen based on survival with the maximum decline in renal function.

### Surgical Procedures

Anaesthesia was induced with 5% sevoflurane and maintained at 2.5–3% in O_2_/N_2_O. Isotonic saline was administered subcutaneously (s.c.) at two different sites (4 mL total) to keep the animals properly hydrated during surgery. This was repeated 24 hours post-operatively. Buprenorphine (Temgesic®; RB Pharmaceuticals Limited, Berkshire, UK) 0.05 mg/kg s.c. was administered to provide sufficient pain relief after surgery. Injections of buprenorphine were repeated 8–12 and 24 hours post-operatively. Subsequently, buprenorphine was administered in the drinking water at 0.75 mg/kg for 24–72 hours post-operatively. The anaesthetic and analgesic regimes were similar for the nephrectomy and the index ischemia operation (rats were not supplemented with injections of isotonic saline after the nephrectomy). The rats were placed on a heating pad and a rectal probe was used to maintain a stable body temperature at 37–38°C. Following a midline incision in the abdominal wall, the left renal artery was carefully dissected to facilitate easy clamp positioning. A non-traumatic vessel clamp was positioned on the renal artery to induce 37 minutes of warm ischemia. To avoid vasospasms in the renal artery, a small drop of lidocaine (10 mg/mL) was administered to the area both prior to dissection and shortly prior to clamp removal. After clamp removal, reperfusion was verified visually. For sham operations, the same steps were performed as mentioned above without renal artery clamping and with only minimal arterial dissection. For the rIC procedures, a part of the abdominal aorta just above the aortic bifurcation was carefully dissected and a non-traumatic clamp was positioned in accordance with the 4×5+5–minute I+R protocol (Fig 1). The abdominal wall was closed in two layers using 4–0 absorbable sutures. Blood was sampled from the tail vein under anaesthesia on day 3 after IRI. Seven days after IRI, the rats were re-anaesthetised, the left kidney was harvested, and blood was sampled from the abdominal aorta. Rats were terminated by cervical dislocation. One rat was supplemented with 4 mL of isotonic saline s.c. due to inadequate oral intake of water 48 hours after IRI.

### Renal Functional Measurements

Plasma creatinine, urea, Na^+^, and urinary creatinine were measured using the Roche Cobas 6000 analyser (Roche Diagnostics, Basel, Switzerland).

### Tissue Handling

After its excision, the kidney was divided into cortex and outer medulla or inner medulla and snap-frozen in liquid nitrogen. Blood samples were collected in EDTA tubes and centrifuged 10 minutes at 3000 × *g*. Kidney, blood, and urine samples were stored at -80°C until use.

### Quantitative Polymerase Chain Reaction

The cortical tissue was homogenised in lysis buffer (Macherey Nagel, Düren, Germany) on a TissueLyser LT (Qiagen, Venlo, Netherlands) for 30 seconds at 1,250 rpm and centrifuged at 1000 × *g* for 10 minutes at 4°C. Total RNA was obtained using an RNA Isolation Kit (Macherey Nagel) in accordance with the manufacturer’s instructions. The RNA concentration and purity were determined spectrophotometrically. The cDNA was synthesised using a RevertAid First Strand cDNA synthesis kit (Thermo Fisher Scientific, Waltham, MA, USA). The quantitative polymerase chain reaction (QPCR) samples were prepared using Maxima SYBR Green QPCR Master Mix (Thermo Fisher Scientific). To generate a standard curve, an equal amount of cDNA from each experimental group was mixed, diluted sequentially, and put into separate wells. The QPCR protocol consisted of 40 cycles of first denaturation (30 seconds at 90°C) and second annealing and synthesis (60 seconds at 60°C). The sequences of the primers used are shown in Table 1.

**Table 1:** Sequences of the primers used for QPCR.

Primer	Sequence
KIM-1	*sense* 5′- CCA CAA GGC CCA CAA CTA TT -3′
	*antisense* 5′- TGT CAC AGT GCC ATT CCA GT -3′
NGAL	*sense* 5′- CAA GTG GCC GAC ACT GAC TA -3′
	*antisense* 5′- GGT GGG AAC AGA GAA AAC GA -3′
IL-1β	*sense 5*′- *CAC AGC AGC ATC TCG ACA AGA -*3′
	*antisense 5*′- *AAG ACA TAG GTA GCT GCC ACA GC -*3′
TNF-α	*sense 5*′- *GCC CTA AGG ACA CCC CTG AGG GAG C -*3′
	*antisense 5*′- *TCC AAA GTA GAC CTG CCC GCA CTC C -*3′
ICAM-1	*sense 5*′- *TCC AAT TCA CAC TGA ATG CC -*3′
	*antisense 5*′- *GTCTGCTGAGACCCCTCTTG* -3′
TGF-β1	*sense 5*′- *TGA GTG GCT GTC TTT TGA CG -*3′
	*antisense 5*′- *TTC TCT GTG GAG CTG AAG CA -*3′
α-SMA	*sense 5*′- *CAT CAT GCG TCT GGA CTT GG -*3′
	*antisense 5*′- *CCA GGG AAG AAG AGG AAG CA -*3′
FN-1	*sense 5*′- *CCG AAT CAC AGT AGT TGC GG -*3′
	*antisense 5*′- *GCA TAG TGT CCG GAC CGA TA -*3′
Collagen-1a	*sense 5*′- *TCA AGA TGG TGG CCG TTA CT -*3′
	*antisense 5*′- *CAT CTT GAG GTC ACG GCA TG -*3′
iNOS	*sense 5*′- *GGG AGC CAG AGC AGT ACA AG -*3′
	*antisense 5*′- *CAT GGT GAA CAC GTT CTT GG -*3′
18S	*sense 5*′- *CAT GGC CGT TCT TAG TTG -*3′
	*antisense 5*′- *CAT GCC AGA GTC TCG TTC -*3′

Sequences of the used primers for QPCR for measuring mRNA expressions. QPCR, quantitative polymerase chain reaction; KIM-1, kidney injury molecule-1; NGAL, neutrophil gelatinase-associated lipocalin; IL-1β, interleukin-1β; TNF-α, tumour necrosis factor-α; ICAM-1, intercellular adhesion molecule-1; TGF-β1, transforming growth factor-β1; α-SMA, α-smooth muscle actin; FN-1, fibronectin-1; iNOS, inducible nitric oxide synthase; 18S, 18S ribosomal RNA

### Western Blot Analysis

The frozen kidney samples were homogenised in radioimmunoprecipitation assay buffer supplemented with Protease Inhibitor Cocktail 2 and 3 (Sigma Aldrich, St. Louis, MO, USA) and a Complete Mini-Protease Inhibitor Tablet (Roche Diagnostics) on the TissueLyser LT. First, a small amount of the supernatant was used to measure protein concentration using a Pierce BCA Protein Assay Kit (Thermo Fischer Scientific) and spectrophotometry (561 nm). Each supernatant was then supplemented with sample buffer (sodium dodecyl sulphate, glycerol, and bromophenol blue) and heated for 15 minutes at 65°C. Subsequently, the samples were loaded on Criterion TGX Stain-Free gels (BioRad, Hercules, CA, USA) and subjected to electrophoresis. The gels were activated using a western blotting imager (ChemiDoc MP; BioRad) and the proteins were transferred to nitrocellulose membranes (Biorad). Total protein level was measured using the western blotting imager as described in the study by Gurtler et al. [14]. Membranes were blocked in 5% skim milk and phosphate buffered saline-Tween (PBS-Tween), washed with PBS-Tween, and incubated overnight at 4°C with primary antibody raised against the target protein (Table 2). Next, the washed membranes were incubated at room temperature for 60 minutes with relevant horseradish conjugated secondary antibodies (polyclonal goat anti-mouse IgG [P447] or polyclonal goat anti-rabbit IgG [P448]; Dako, Glostrup, Denmark). Finally, the antibodies were visualised using an enhanced chemiluminescence system (Amersham Pharmacia Biotech, Piscataway, NJ, USA) and the western blotting imager.

**Table 2:** Primary antibodies used for western blotting.

Antibody	Manufacturer	Product code	Species
CAT	Abcam, Cambridge, UK	Ab76024	Rabbit
MnSOD	Millipore, Darmstadt, Germany	06–984	Rabbit
HSP32	Enzo Life Science, Farmingdale, NY, USA	ADI-SPA-896	Mouse
pHSP27	Abcam, Cambridge, UK	Ab5594	Rabbit
HSP70	Cayman Chemical, Ann Arbor, MI, USA	10011421	Mouse
pAkt (Ser473)	Cell Signaling, Danvers, MA, USA	4060	Rabbit
Akt	Cell Signaling, Danvers, MA, USA	4691	Rabbit
pERK1/2 (Thr202/Tyr204)	Cell Signaling, Danvers, MA, USA	4370	Rabbit
ERK1/2	Cell Signaling, Danvers, MA, USA	4695	Rabbit

Primary antibodies were raised against the following proteins: CAT, catalase; MnSOD, manganese superoxide dismutase; HSP32, heat shock protein 32; pHSP27, phosphorylated heat shock protein 27; HSP70, heat shock protein 70; pAkt (Ser473), phosphorylated protein kinase B on serine-473; Akt, protein kinase B; pERK1/2, phosphorylated extracellular signal-regulated kinase 1/2 on threonine-202 and tyrosine-204; ERK1/2, extracellular signal-regulated kinase ½.

### Statistical Analysis

Data are expressed as mean values ± standard error of the mean (SEM). Normality was confirmed by a Q-Q plot. When necessary, data were ln-transformed. For normally distributed data, one-way analysis of variance (ANOVA) with Bonferroni’s multiple comparison post-test was used to test for significant differences. The Kruskal-Wallis test with Dunn’s multiple comparisons post-test was used for non-Gaussian distributed data. A paired t-test was used to test for significant differences between CrCl (or pCr) measured at different time intervals within each experimental group. Sample size estimation is based on observations in the study by Fuller et al. 2005 [15]. In this study, local IPC of the kidney (15 minutes of ischemia followed by 10 minutes of reperfusion) reduced pCr by 67% 6 days after IRI. We assumed that the protective effect of rIC was of a lesser magnitude and hypothesized that it would lead to a 33% reduction in pCr at day 7. The standard deviation (SD) in this study was approximately 25% of the mean pCr which we used to estimate a SD in our IR+rIPerC group. Based on these assumptions and normal distribution of the data, the required sample size was calculated using the sampsi function in STATA (sampsi 2.7 1.8, sd1(0.7), sd2(0.5), power = 0.9, alpha(0.05)). This resulted in a required sample size of n = 10. We expected SD to be lower in the sham group and reduced the number of animals (n = 7). Data were analysed using STATA version IC/13.1 for Windows (StataCorp LP, College Station, TX, USA) and GraphPad Prism version 6.05 for Windows (GraphPad Software, San Diego, CA, USA). P values < 0.05 were considered statistically significant.

## Results

### Use of rIC did not Protect against IRI-Associated AKI

**Fig 2:**
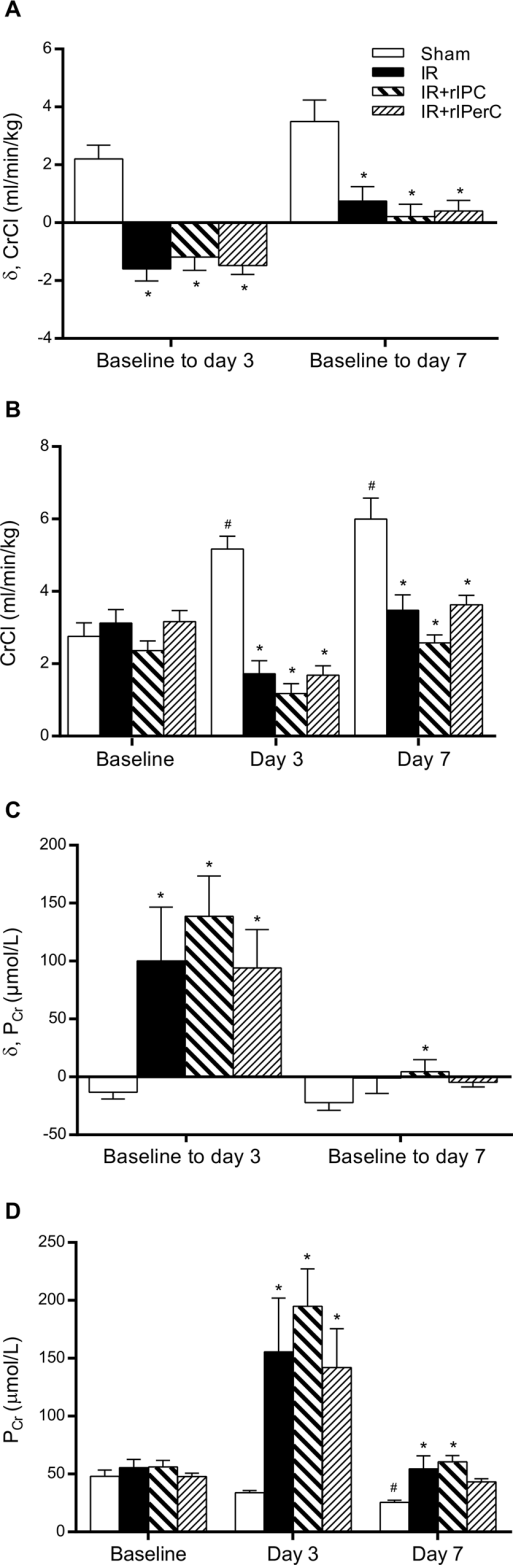
Changes in creatinine clearance (CrCl) rate and plasma creatinine (pCr) after ischemia-reperfusion injury. A) Presented as the difference (δ) between CrCl rate at baseline (prior to IRI) and CrCl rate at 3 and 7 days after IRI. B) The absolute value for the CrCl rate at the different time intervals. C) The difference between pCr at baseline and pCr at 3 and 7 days after IRI. D) The absolute value for the pCr at the different time intervals. All values are expressed as mean ± SEM. Group means were compared using one-way analysis of variance followed by Bonferroni’s multiple comparison post-test or Kruskal-Wallis test with Dunn's multiple comparisons post-test. *P < 0.05 vs. sham. A paired t-test was used to test for significant differences between CrCl or pCr measured at 3 and 7 days after IRI and the baseline value within the sham group #P < 0.05. Number of animals: sham (n = 6), ischemia-reperfusion (IR; n = 8–10), IR and remote ischemic preconditioning (IR+rIPC; n = 10), IR and remote ischemic perconditioning (IR+rIPerC; n = 10).

All animals survived until termination of the study. IRI was associated with a significant decrease in CrCl rate from baseline to day 3 compared with the sham group (Fig 2A). The decline in CrCl rate was similar in the IR (δ: -1.59 mL/min/kg [-2.55 to -0.64]), IR+rIPC (δ: -1.19 mL/min/kg [-2.22 to -0.15], *p* > 0.9999), and IR+rIPerC (δ: -1.48 mL/min/kg [-2.17 to -0.79], *p* > 0.9999) groups at day 3. At day 7, all groups exposed to IRI recovered a CrCl rate similar to their respective baseline values (IR group, δ: 0.74 mL/min/kg [-0.45 to 1.94], *p* = 0.18; IR+rIPC group, δ: 0.21 mL/min/kg [-0.75 to 1.17], *p* = 0.63; and IR+rIPerC group, δ: 0.41 mL/min/kg [-0.43 to 1.25], *p* = 0.30) (Fig 2B and Table 3). Of note, the sham group displayed a significantly increased CrCl rate from baseline to study end (δ: 3.24 [1.66 to 4.82] mL/min/kg, *p =* 0.0052). The pCr results are presented in Fig 2C and 2D and correspond to the results based on the CrCl rate.

**Table 3:** Physical, plasma, and urine parameters.

** **		**Baseline**				
**Parameter**		Sham	IR	IR+rIPerC	IR+rIPC	
BW, g		258 ± 2.9	262 ± 2.9	268 ± 3.1	261 ± 2.2	
Intake_water_, mL		46 ± 4.8	40 ± 2.6	44 ± 4.2	54 ± 2.8	
Intake_food_, g		34 ± 8.9	19 ± 1.4	28 ± 8.9	19 ± 0.9	
CrCl, mL/min/kg		2.8 ± 0.4	3.1 ± 0.4*	3.2 ± 0.3*	2.4 ± 0.3*	
P_cr,_ μmol/L		48 ± 5.4	56 ± 7.0	48 ± 2.8	56 ± 5.6	
P_urea,_ mmol/L		10 ± 0.8	11 ± 1.2*	12 ± 0.7*	12 ± 0.7*	
		**Day 3**				
**Parameter**		Sham	IR	IR+rIPerC	IR+rIPC	
BW, g		260 ± 5.1	249 ± 4.3	249 ± 3.7	241 ± 2.3*	
Intake_water_, mL		35 ± 4.0	34 ± 3.3	32 ± 3.4	35 ± 2.5	
Intake_food_, g		15 ± 0.7	9 ± 1.0*	8 ± 1.3*	7 ± 0.5*	
CrCl, mL/min/kg		5.2 ± 0.4	1.7 ± 0.4*	1.7 ± 0.3*	1.2 ± 0.3*	
P_cr,_ μmol/L		34 ± 1.8	156 ± 46.4*	142 ± 33.7*	195 ± 32.3*	
P_urea,_ mmol/L		9 ± 0.2	28 ± 6.3*	26 ± 5.3*	34 ± 5.1*	

		**Day 7**				
**Parameter**		Sham	IR	IR+rIPerC	IR+rIPC	
BW, g		251 ± 4.2	241 ± 5.1	245 ± 4.0	234 ± 1.9	
Intake_water_, mL		27 ± 3.0	41 ± 3.3*	40 ± 3.2	42 ± 3.6*	
Intake_food_, g		18 ± 0.7	16 ± 1.2	17 ± 1.0	15 ± 0.8	
KW, g/100 g BW		5 ± 0.2	11 ± 1.0*	11 ± 1.1*	12 ± 0.7*	
CrCl, mL/min/kg		6.0 ± 0.6	3.5 ± 0.4*	3.6 ± 0.3*	2.8 ± 0.2*	
P_cr,_ μmol/L		25 ± 2.0	55 ± 11.3*	43 ± 2.7	61 ± 5.4*	
P_urea,_ mmol/L		6 ± 0.4	15 ± 3.6*	12 ± 0.9*	16 ± 1.8*	

Values are presented as means ± SEM. Group means were compared using either one-way analysis of variance followed by Bonferroni’s multiple comparison post-test or Kruskal-Wallis test with Dunn's multiple comparisons post-test.

*P < 0.05 vs. sham.

BW, body weight; Intake_water_, 24-hour water intake; Intake_food_, 24-hour food intake; KW, kidney weight; CrCl, creatinine clearance; P_cr_, plasma creatinine; P_urea_, plasma urea; U_output_, urinary output; FE_Na_, fractional excretion of sodium; IR, ischemia-reperfusion; rIPerC, remote ischemic perconditioning; rIPC, remote ischemic preconditioning.

**Fig 3:**
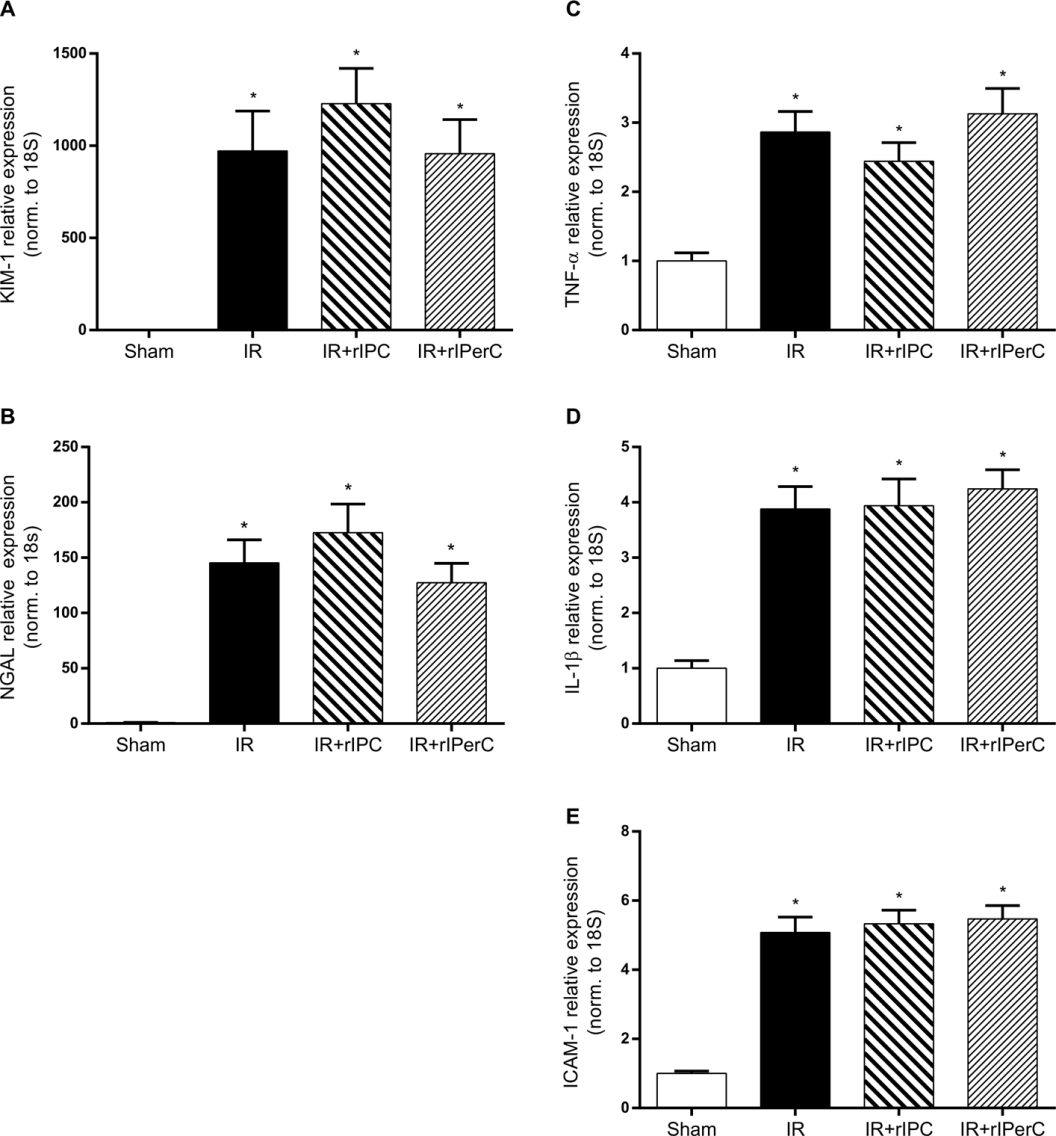
Effect of ischemia-reperfusion injury and remote ischemic conditioning on the renal expression of injury and inflammatory markers after 7 days of reperfusion. A) Kidney injury molecule-1 (KIM-1). B) Neutrophil gelatinase-associated lipocalin (NGAL). C) Tumour necrosis factor-α (TNF-α). D) Interleukin-1β (IL-1β). E) Intercellular adhesion molecule-1 (ICAM-1). Bars represent mRNA levels by quantitative polymerase chain reaction in the left renal cortex/outer medulla normalised to 18S mRNA. All values are expressed as mean ± SEM. Group means were compared using one-way analysis of variance followed by Bonferroni’s multiple comparison post-test. *P < 0.05 vs. sham. Number of animals (all markers): sham (n = 7), ischemia-reperfusion (IR; n = 10), IR and remote ischemic preconditioning (IR+rIPC; n = 10), IR and remote ischemic perconditioning (IR+rIPerC; n = 10).

Both KIM-1 and NGAL mRNA expression increased in response to IRI at day 7 (Fig 3A and 3B), reflecting damage to both proximal and distal tubules, and rIC did not change these levels. Overall, the functional measurements and tissue damage markers suggest that rIC did not provide a protective effect against IRI at days 3 and 7.

IRI was associated with a significant inflammatory response when measured by renal mRNA expression levels of tumour necrosis factor-α, interleukin-1β, and intercellular adhesion molecule-1 and compared with sham (*p* = 0.0001) (Fig 3C–3E). Neither rIC procedure alleviated the inflammatory response.

### IRI and Recovery was Associated with Cytoprotective Protein and Fibrosis Marker Up-regulation

**Fig 4:**
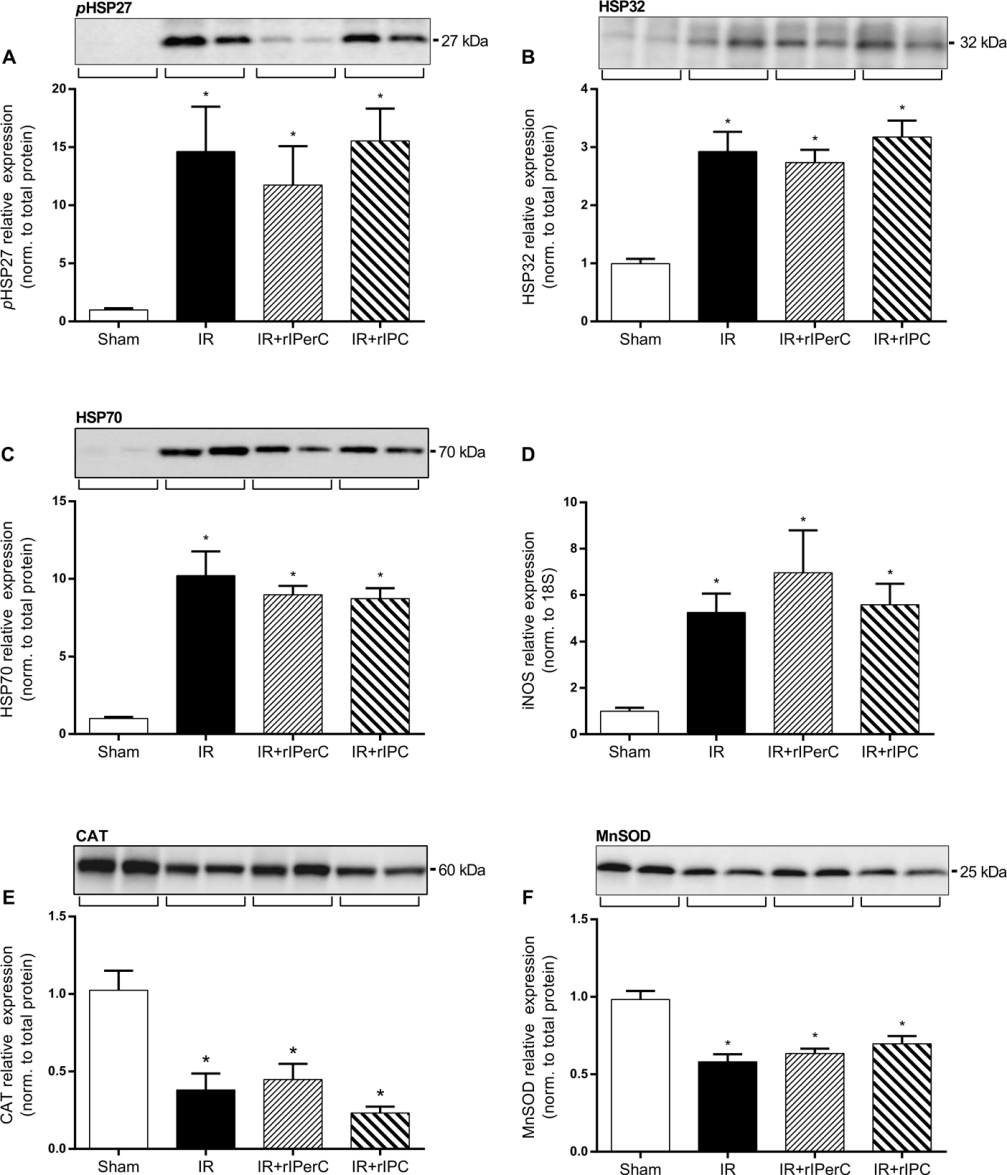
Effect of ischemia-reperfusion injury and remote ischemic conditioning on heat shock protein and anti-oxidant levels and inducible nitric oxide synthase (iNOS) expression after 7 days of reperfusion. A) Phosphorylated heat shock protein 27 (pHSP27). B) Heat shock protein 32 (HSP32). C) Heat shock protein 70 (HSP70). E) Catalase (CAT). F) Manganese superoxide dismutase (MnSOD). Bars represent protein levels by western blotting normalised to total protein. All values are expressed as mean ± SEM. Number of animals (all proteins): sham (n = 7), ischemia-reperfusion (IR; n = 10), IR and remote ischemic preconditioning (IR+rIPC; n = 10), IR and remote ischemic perconditioning (IR+rIPerC; n = 10). Representative western blots (n = 2 in all groups). D) Inducible nitric oxide synthase (iNOS). Bars represent mRNA levels by quantitative polymerase chain reaction in left renal cortex/outer medulla normalised to 18S mRNA. All values are expressed as mean ± SEM. Group means were compared using one-way analysis of variance followed by Bonferroni’s multiple comparison post-test. *P < 0.05 vs. sham. Number of animals: sham (n = 7), IR (n = 10), IR+rIPC (n = 10), IR+rIPerC (n = 10).

The active phosphorylated form of HSP27 and the total protein levels of HSP32 and HSP70 were significantly up-regulated in the groups exposed to IRI compared with the sham group. No significant effect of rIPC or rIPerC was detected (Fig 4A–4C). The iNOS level was significantly up-regulated in all groups compared with the sham group (*p* < 0.0001), with no effect of rIPC or rIPerC (Fig 4D). IRI was associated with a significant reduction in the levels of the anti-oxidant enzymes CAT and MnSOD (p = 0.004, p < 0.0001, respectively), whereas rIC did not affect their levels compared to IRI alone (Fig 4E and 4F).

**Fig 5:**
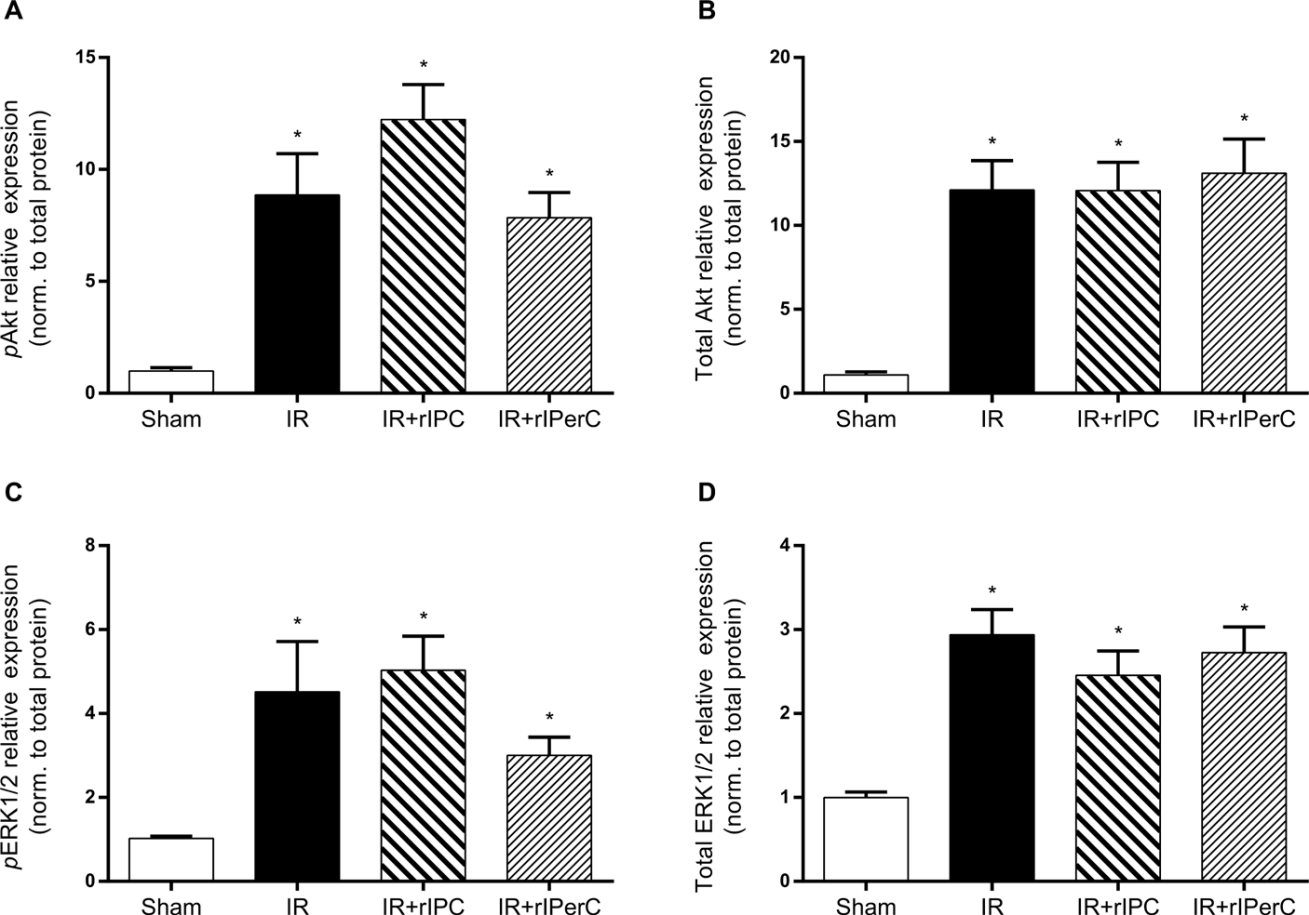
Effect of ischemia-reperfusion injury and remote ischemic conditioning on protein expression levels of phosphorylated and total protein kinase B (Akt) and extracellular signal-related kinase 1/2 (ERK1/2) protein levels. A) Phosphorylated protein kinase B (pAkt). B) Total protein kinase B (Akt). C) Phosphorylated ERK1/2 (pERK1/2). D) Total ERK1/2. Bars represent protein level by western blotting normalised to total protein. All values are expressed as mean ± SEM. Group means were compared using one-way analysis of variance followed by Bonferroni’s multiple comparison post-test. *P < 0.05 vs. sham. Number of animals: sham (n = 7), ischemia-reperfusion (IR; n = 10), IR and remote ischemic preconditioning (IR+rIPC; n = 10), IR and remote ischemic perconditioning (IR+rIPerC; n = 10).

The expressions of active phosphorylated MAPK pAkt and pERK1/2 were markedly up-regulated after IRI compared with sham treatment (both *p* < 0.0001) (Fig 5A–5C). There was no additional change in these levels in relation to rIPC or rIPerC. Total protein levels of Akt and ERK1/2 were also significantly higher in all groups exposed to IRI compared with the sham group (Fig 5B–5D).

**Fig 6:**
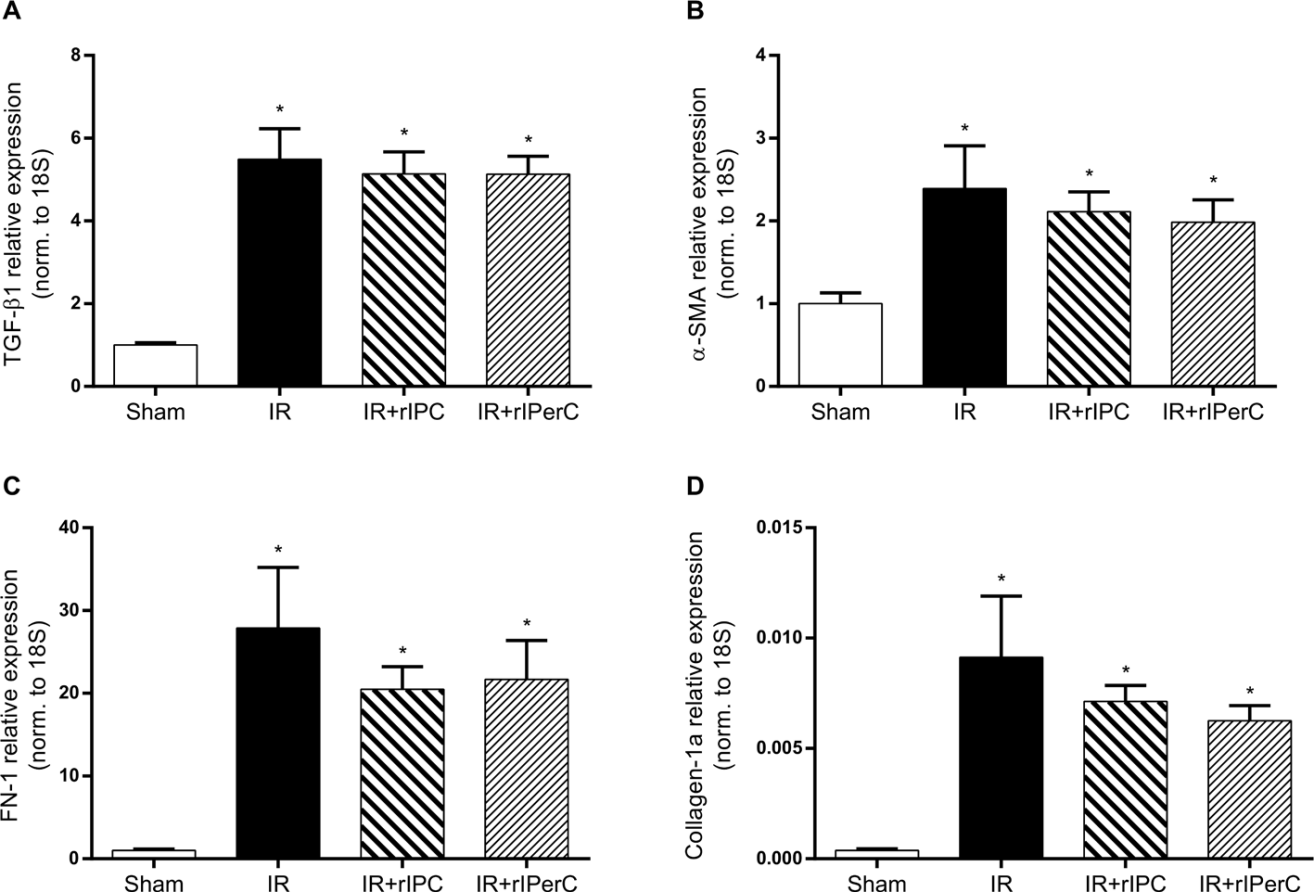
Effect of ischemic-reperfusion injury and remote ischemic conditioning on pro-fibrotic mediator and fibrosis marker expression. A) Transforming growth factor-β1 (TGF-β1). B) α-smooth muscle actin (α-SMA). C) Fibronectin-1 (FN-1). D) Collagen-1a. Bars represent mRNA levels by quantitative polymerase chain reaction in the left renal cortex/outer medulla normalised to 18S mRNA. All values are expressed as mean ± SEM. Group means were compared using one-way analysis of variance followed by Bonferroni’s multiple comparison post-test. *P < 0.05 vs. sham. Number of animals: sham (n = 7, n = 5 for FN-1), ischemia-reperfusion (IR; n = 10), IR and remote ischemic preconditioning (IR+rIPC; n = 10), IR and remote ischemic perconditioning (IR+rIPerC; n = 10).

IRI was associated with a significant increase in the expression of the pro-fibrotic mediators TGF-β1 and α-SMA and the fibrosis markers FN-1 and collagen-1a compared with sham (*p* < 0.0001, *p* = 0.0149, *p* < 0.0001, and *p* = 0.0175, respectively) (Fig 6). No significant effect of rIPC or rIPerC on the expression of any of the measured pro-fibrotic mediators or fibrosis markers was observed.

## Discussion

In this study, we demonstrated that the kidney to some extent recovers function within 7 days after 37 minutes of warm ischemia in a unilateral nephrectomy rat model. Recovery of renal function was associated with a significant up-regulation of pAkt, pERK1/2, pHSP27, HSP32, and HSP70. The iNOS mRNA expression level was increased significantly 7 days after IRI as well, while the levels of the anti-oxidant enzymes CAT and MnSOD were diminished. IRI was associated with persistent injury, inflammation, and an ongoing pro-fibrotic response after 7 days. In contrast to our hypothesis, we did not observe an effect of any of the two rIC strategies on kidney function, cytoprotective protein expression, or injury, inflammation, or fibrosis indicators.

### Lacking Effect of rIC

To our surprise, both rIC algorithms failed to provide renoprotection and did not attenuate the ongoing inflammatory response and pro-fibrotic process in the kidney following IRI. This result is in line with earlier experimental studies reporting a lacking effect of rIC [16–20]. In our setup, at least four important factors could have affected the tissue protection potentially offered by rIC: the rIC protocol/IRI model, the anaesthesia and analgesic protocol, vessel dissection, and/or prior nephrectomy [21]. First, the optimal algorithm and anatomical site of applying the rIC remains to be established in renal IRI models (timing, duration, and number of IR cycles), and our approach might have been insufficient. Song et al. reported a renoprotective effect of rIPC applied as three cycles of 8 minutes of ischemia and 5 minutes of reperfusion by clamping of the anterior mesenteric artery prior to 45 minutes of unilateral renal ischemia [22]. We chose to apply the rIC stimulus on a large tissue mass and used a fractionated rIC algorithm in accordance with the study by Wever et al. [23]. Second, both sevoflurane and buprenorphine in particular could have interfered with the outcome of rIC in our study. It was previously demonstrated that volatile anaesthetics generate a potent conditioning stimulus similar to IC [24–27]. In addition, buprenorphine producing both partial agonistic effects at the μ-opioid receptors, agonistic effects at the δ-opioid receptors, and antagonistic effects at κ-opioid receptors could have either blunted or blocked the effect of rIC [28,29]. We have located 3 experimental renal rIC studies in which buprenorphine has been used as a perioperative analgesic [16,20,22]. Timing of the buprenorphine administration may be critical. Thus, to counteract the effect of rIC, buprenorphine must have the maximal blocking effect if it is administered prior to the applied rIC stimulus. In the studies, buprenorphine was administered either at induction of anesthesia [16], “intraoperatively” [20] or immediately after surgery [22]. Of note, only the latter study found an effect of rIC. It must be emphasized that the studies are quit heterogenic in several aspects. In regard to sevoflurane, we have not identified any renal rIC studies using this agent. In contrast, the related agent isoflurane is widely used both in small and large animal studies, where renoprotective effects of rIC is reported in some [23,29–32] but not all [16,20]. Sevoflurane in combination with buprenorphine was chosen since they provide optimal anaesthetic and analgesic effects in combination with a safe profile when compared with i.v. anaesthetics [33]. Third, coincidental local IPC or IPoC of the kidney due to vasospasm during the dissection could have blunted the effect of rIC. Precautions were taken to avoid vasospasms in the renal artery prior to clamp positioning by careful dissection and the local use of lidocaine (also used in our renal transplantation procedure in rats). However, besides its vasorelaxant property, lidocaine is capable of minimizing IRI. This has been demonstrated in various organs and tissues (kidney, brain, heart lung, intestine, free skin flap), across different animal species and both in relation to in situ IRI and IRI in transplants [34–39]. Fourth, we speculate that the nephrectomy performed prior to IRI and rIC may have blunted the effects of rIC. Studies have reported that the removal of a non-ischemic kidney protects the contralateral kidney against IRI [40–42]. This phenomenon could potentially be mediated by similar mechanisms as IC, and we may have been performing our experiment in the late window of protection after remote preconditioning (kidney on kidney). Indeed, we observed a significant increase in the CrCl rate (and a corresponding decrease in pCr) of the nephrectomised, sham-operated rats from baseline to study end, indicating a compensatory growth and function of the remaining kidney. Overall, further studies must address these issues.

### Mediators of Kidney Recovery after IRI

A number of studies have shown that the kidney may be dependent on MAPK phosphorylation to provide immediate defence against IRI since phosphorylation is increased early after reperfusion [43,44]. This may also be important for the recovery phase [12,43–46], although studies have reported conflicting results [47–51]. This is possibly due to the diversity of the signalling cascades and cellular processes influenced by MAPK. Our data demonstrated Akt and ERK1/2 activation in response to 37 minutes of ischemia followed by 7 days of reperfusion. This activation occurred simultaneously with the recovery of renal function from day 3 to day 7. This is consistent with a study by Jang HS et al. in which a sustained activation of ERK1/2 was observed up to 9 days following 30 minutes of unilateral renal ischemia in mice. Inhibition of ERK1/2 from day 1 after reperfusion resulted in increased tissue damage and a decreased proliferation of tubular epithelial cells, while interstitial cell proliferation, extracellular matrix deposition, and TGF-β1 expression levels were increased [12]. It was also demonstrated that pAkt inhibition 4 hours prior to 35 minutes of bilateral renal ischemia in mice reduced kidney function, increased histopathological damage, and decreased tubular cell proliferation (24–48 hours after reperfusion) [46]. Taken together, these findings indicate that ERK1/2 and Akt activation plays a significant role in the recovery phase following IRI. However, the precise role of MAPK and the importance of when these are active in the course of renal IRI have yet to be established.

HSP27, HSP32, and HSP70 are well-established cytoprotective proteins with diverse effects that counteract IRI-related damage, including anti-apoptotic [52], anti-oxidant [53], and anti-inflammatory [54] effects as well as the ability to refold damaged proteins [55] and stabilise the cytoskeleton [56]. The expression and activation of these proteins make them sensitive to IRI (28–33). Of note, increased HSP27 protein expression has been detected up to 12 weeks after 30 minutes of ischemia in the mouse kidney [57]. The results of the present study show that IRI not only increases HSP27phosphorylation but also increases HSP32 and HSP70 expression levels 7 days after reperfusion. As discussed in the study by Park et al. [57], this prolonged activation may be caused by persistent ongoing inflammatory activity, reactive oxygen species generation, and epithelial cell de-differentiation. In the present study, we confirmed that this connection may be responsible for not only the increased HSP expression levels but also for KIM-1, NGAL, and iNOS regulation. The up-regulation of iNOS and the following increase in nitric oxide (NO) might have contributed to the functional recovery that we observed [58,59]. On the other hand, excess NO would cause further tissue damage [59,60]. Interestingly, the increased expression of KIM-1 and NGAL may not only serve as markers of IRI-induced tissue damage. An in vivo study showed that the up-regulation of KIM-1 on the surface of tubular epithelial cells after IRI enables these cells to clear apoptotic and necrotic cells via phagocytosis [61]. However, chronic KIM-1 expression has also been linked to renal interstitial fibrosis [62]. In particular, NGAL is capable of inhibiting apoptosis and stimulating cell proliferation in proximal tubule cells [63]. In summary, our results suggest that selected cytoprotective proteins including MAPK, HSPs, iNOS, NGAL, and KIM-1 may participate in the recovery phase after IRI. Further research is necessary to clearly establish the exact role of and possible interplay between these mediators.

### Lacking Effect of rIC on Molecular Targets

In the present study, we did not observe any effect of rIC on pERK1/2 and pAkt. Regarding pERK1/2, this could be due to our use of rIC instead of local IC [43,47], rIPC/rIPerC instead of local IPoC (ischemic post-conditioning) [43], and the use of rats instead of mice [47]. A previous study examining pAkt and total Akt expression in the kidney in response to rIPC differed from ours in terms of: 1) the remote site used for IC (the liver instead of the hind limb/pelvic organs), 2) the algorithm (a single cycle of 10 minutes of ischemia followed by 10 minutes of reperfusion vs. four cycles of 5 minutes of ischemia and 5 minutes of reperfusion), 3) anaesthetic agent (intraperitoneal pentobarbital vs. inhaled sevoflurane/N_2_O), 4) duration of unilateral renal ischemia (30 minutes vs. 37 minutes), and 5) sampling time of pAkt and total Akt (15 minutes vs. 7 days) [64]. A number of studies have investigated the effect of local IPC or IPoC on pAkt in both unilateral and bilateral renal IRI models in which the duration of ischemia ranged from 30 to 60 minutes, using various IPC and IPoC algorithms, and earlier sampling of pAkt (from 5 minutes to 24 hours after renal ischemia) [43,47,50,65].

To our knowledge, the current study is the first to examine the renal expression of HSPs in relation to rIC. Earlier studies reported increased HSP levels following local IPC (28–33) and local IPoC (34, 35). Here we did not detect any increased levels of HSPs after rIC. In addition, no effect of rIC on the expression of iNOS, an important mediator of delayed local IPC [47,57] and rIC [64], was observed.

In the present study, rIC did not change CAT and MnSOD expressions, a finding that is in contrast to those of other studies of local IC [66–68] and rIC [22,69]. This may be related to differences in rIC procedures, the assays used to measure CAT and MnSOD (measurement of enzyme activity vs. protein levels used in our study), and sampling times. Sedaghat et al. reported a significant increase in CAT and SOD activity 24 hours after 45 minutes of unilateral renal ischemia in Sprague-Dawley rats that was associated with four cycles of 5 minutes of ischemia and 5 minutes of reperfusion (rIPerC) on the left femoral artery (compared to the IR group) [69]. In the study by Song et al., rIPC applied as three cycles of 8 minutes of ischemia and 5 minutes of reperfusion on the anterior mesenteric artery protected against IRI after 45 minutes of unilateral ischemia in Wistar rats [22]. The use of rIPC also resulted in significantly increased CAT activity at 2 and 24 hours after IRI and in SOD activity at 2 hours after IRI compared with IRI without rIC. Taken together, in our setup, rIC did not have any impact on the proteins that are suggested to be involved in the immediate protective effects against renal IRI or in the recovery phase after IRI.

### Limitations

The main draw backs of this study include issues concerning randomization, bias, blinding, and variability in the results. In the course of the study, we added a new group, i.e. the IR+rIPC group, to add valuable information to the study and to the research area. Unfortunately, this introduced selection bias and an increased risk of type 1 error. We performed the study in two rounds each consisting of two consecutive surgery days. Only in the second round animals were subjected to IR+rIPC. Thus, each animal did not have an equal chance to be allocated to each of the treatment groups overall. Nevertheless, animals were randomly allocated to all 4 groups in the second round, thereby minimizing the threat to the internal validity of the study. The scientists (CKL and MLVK) performing the operations (and aware of which treatment each animal had received) also monitored the animals’ health, handled them to administer analgesics, to collect urine in the metabolic cages and to sample blood etc. In this way, some risk of performance bias exists. In terms of detection bias, QPCR and Western blotting were performed mainly by skilled laboratory technicians, who were blinded to the intervention. However, CKL did also run some of the analyses, assessed the results and was not blinded. Of note, these analyses were performed, analyzed and assessed meticulously and in a uniform and standardized way.

We did detect a large variability in the functional parameters. This could be related partly to urine collection in the metabolic cages. Differences in the degree of stress exposition, temperature, incomplete collection of all excreted urine, habituation and food and water intake (which we did observe) could account for some of the variability observed. Importantly, some of these factors could aggravate the IRI, but could also add imprecision to the CrCl measurements. Although temperature was kept constant during surgery, even slight variation could result in differences in the severity of renal IRI [70].

## Conclusions

In summary, kidney recovery 7 days after 37 minutes of unilateral ischemia was associated with the up-regulation of established cytoprotective HSP levels but also persistent inflammatory and pro-fibrotic activities. Continuous ERK1/2 and Akt activation as well as increased iNOS expression may be involved in the repair process after IRI, which requires further clarification in future studies. No protective effects were observed in response to rIPC or rIPerC, a phenomenon that may be related to the contralateral nephrectomy at day -7, choice of rIC protocol/IRI model, or perioperative confounding factors remaining to be elucidated. These results will contribute to the design of future studies with the important aim of preventing kidney IRI.
